# Evaluation of *Pseudomonas* sp. for its multifarious plant growth promoting potential and its ability to alleviate biotic and abiotic stress in tomato (*Solanum lycopersicum*) plants

**DOI:** 10.1038/s41598-020-77850-0

**Published:** 2020-12-01

**Authors:** Sangeeta Pandey, Shikha Gupta

**Affiliations:** Amity Institute of Organic Agriculture, Amity University Uttar Pradesh, Sector 125, Noida, Uttar Pradesh 201313 India

**Keywords:** Microbiology, Plant sciences

## Abstract

1-Aminocyclopropane-1-carboxylate (ACC) deaminase activity is one of the most beneficial traits of plant growth promoting (PGP) rhizobacteria responsible for protecting the plants from detrimental effects of abiotic and biotic stress. The strain S3 with ACC deaminase activity (724.56 nmol α-ketobutyrate mg^−1^ protein hr^−1^) was isolated from rhizospheric soil of turmeric (*Curcuma longa*), a medicinal plant, growing in Motihari district of Indian state, Bihar. The halotolerant strain S3, exhibited optimum growth at 8% (w/v) NaCl. It also exhibited multiple PGP traits such as indole acetic acid production (37.71 μg mL^−1^), phosphate solubilization (69.68 mg L^−1^), siderophore, hydrocyanic acid (HCN) and ammonia production as well as revealed antagonism against *Rhizoctonia solani*. The potential of isolated strain to alleviate salinity stress in tomato plants was investigated through pots trials by inoculating strain S3 through-seed bacterization, soil drenching, root dipping as well as seed treatment + soil drenching. The strain S3 inoculated through seed treatment and soil drenching method led to improved morphological attributes (root/shoot length, root/shoot fresh weight and root/shoot dry weight), photosynthetic pigment content, increased accumulation of osmolytes (proline and total soluble sugar), enhanced activities of antioxidants (Catalase and Peroxidase) and phenolic content in salt stressed tomato plants. The biochemical characterisation, FAMEs analysis and 16S rRNA gene sequencing revealed that strain S3 belongs to the genus *Pseudomonas*. The overall findings of the study revealed that *Pseudomonas* sp. strain S3 can be explored as an effective plant growth promoter which stimulate growth and improve resilience in tomato plants under saline condition.

## Introduction

The population across the world is increasing exponentially; it could reach 9.1 billion globally in the year 2050. However, production of food grains is insufficient to meet requirements of increasing population^1,2^. Among all factors which are responsible for low production, a wide range of biotic and abiotic challenges such as drought, flooding, salinity, heat, cold, exposure to heavy metals as well as pathogen attack limits the crop performance and agronomic yield^3^. Salinity is the major environmental issue challenging the agriculture sector to produce enough food to meet the growing demands of population^4,5^. It is estimated that more than 800 million hectares of land is affected by salinity throughout the world due to irrigation with saline water, excessive usage of chemical based fertilizers, high temperature and scanty precipitation resulted in loss of cultivated soil^6^ (FAO,2008).

Salinity causes excessive accumulation of Na + ions, generating ion imbalance and reactive oxygen species (ROS) in plants resulting in ion toxicity, cellular toxicity, osmotic stress and oxidative stress^7,8^. Apart from oxidative stress, salinity increases the levels of 1-aminocyclopropane-1-carboxylic acid (ACC) and its product, stress hormone known as ethylene^9^. Ethylene is a gaseous hormone involved in regulating various metabolic processes of plants. It is directly involved in developmental processes of plants like germination of seed, root hair development, leaf senescence and abscission and fruit ripening^10^. It has been reported that the excessive salt concentration in the rhizosphere has detrimental effects on the early growth stages of seedling development and thus reduces the rate of seed germination and vegetative growth parameters of various crops^11–13^. In addition to this, the stomatal conductance, chlorophyll content and therefore the photosynthetic machinery were adversely affected in salinity stress condition which challenges the development of sustainable agriculture^14^.

The application of plant growth promoting rhizobacteria (PGPR) with ACC deaminase producing ability in agriculture is an effective strategy, alternative to chemical-based fertilizers, to reduce stress induced ethylene level and its detrimental effects manifested on plants through irreversible breakdown of its precursor, ACC into ammonia and α-ketobutyrate^15^. The soil associated ACC deaminase expressing bacterial strains colonize the roots zone of plants and confer stress resilience in plants through various mechanisms such as indoleacetic acid (auxin) production, siderophore production, solubilization of insoluble metal complexes like phosphorous and zinc, production of ammonia and hydrocyanic acid (HCN) resulting in enhancement of plant growth and development^16^. In addition to this, ACC deaminase containing bacterial strains also possessed an important characteristic of suppressing the growth of several phytopathogenic bacterial and fungal strains and providing resistance to plants against plant pathogens^17^.

In this background, the present study was designed to isolate ACC deaminase producing bacterial strain from rhizospheric soil of medicinally important, turmeric plant (*Curcuma longa*) and to assess its stress tolerance potential in tomato (*Solanum lycopersicum*) plants under saline (100 mM NaCl) conditions. The strain was also functionally characterized for other plant growth promoting traits such as IAA production, phosphate solubilization, siderophore and HCN production under in vitro conditions. Besides this, the biocontrol potential of ACC deaminase producing strain against fungal pathogen, *Rhizoctonia solani* was also determined. The strain was identified phenotypically through fatty acid methyl ester (FAME) analysis method and phylogenetically characterized as *Pseudomonas* sp. strain S3 based on 16S rRNA gene sequencing. Through pot experiments, we evaluated the effect of salinity stress and bacteria inoculation in terms of morphological growth parameters (root/shoot length, fresh and dry biomass of root/shoots), production of stress induced ethylene, accumulation of proline and total soluble sugars, production of total phenolics and antioxidant enzymes (Catalase and Peroxidase), chlorophyll and carotenoid content. Furthermore, we also assessed and compared the effect of different methods of bacterial inoculation to plants–seed bacterization, soil drenching, root dipping as well as combination of seed treatment and soil drenching on growth of tomato plants.

## Results

### Biochemical characterization, FAME analysis and molecular identification of ACC utilizing test isolate

In the present study 10 distinct bacterial strains from turmeric (*Curcuma longa*) rhizosphere were selected after enrichment on LB agar growth medium. Based on ACC utilizing ability, out of 10 bacterial isolates tested, only isolate S3 was able to grow on DF minimal salt medium supplemented with 3 mM ACC as sole nitrogen source instead of (NH_4_)_2_SO_4._

**Figure 1:**
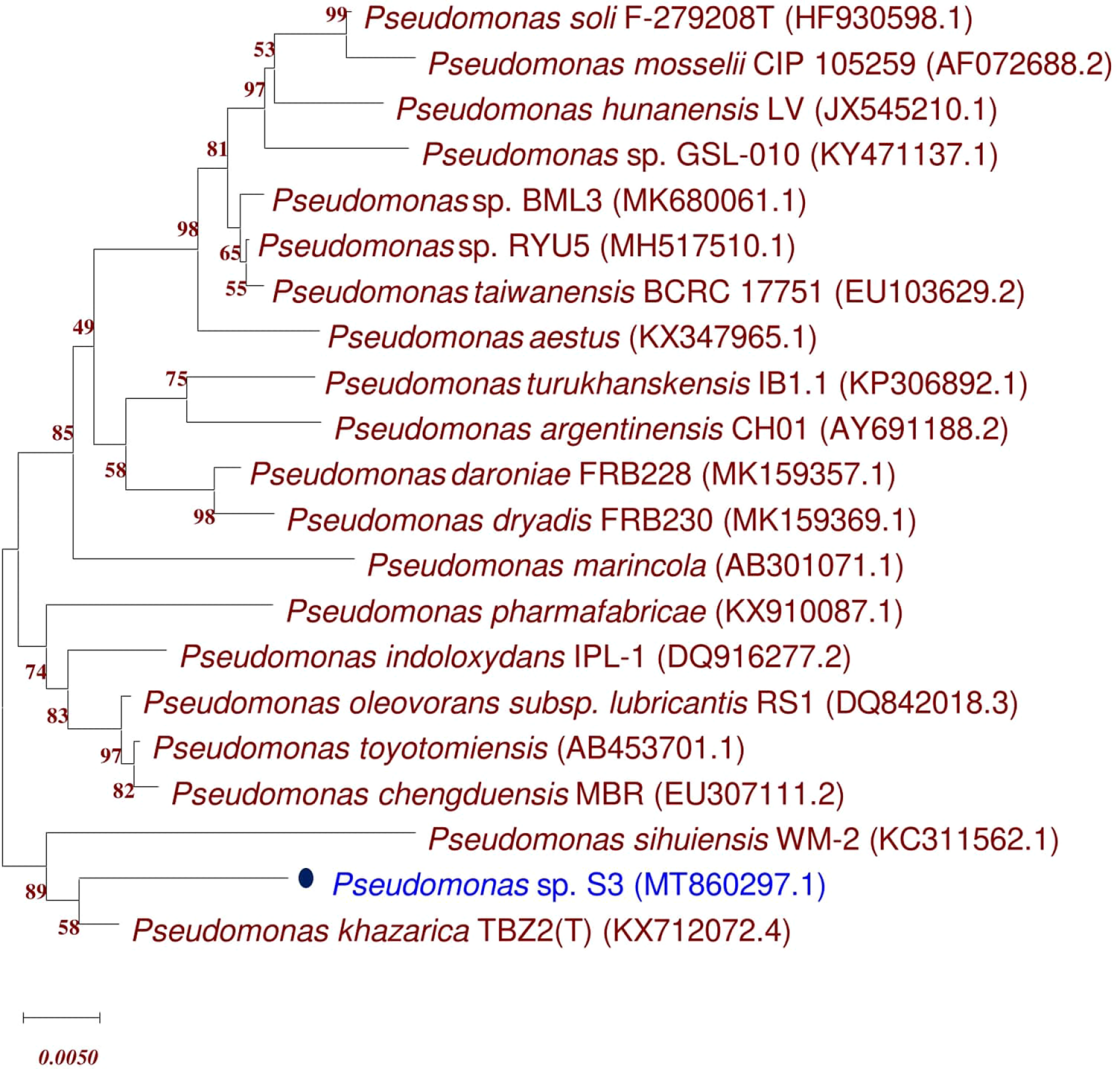
Phylogenetic tree based on a partial 16S rRNA nucleotide sequences showing the position of strain S3 with respect to other related taxa was constructed with Neighbour joining method using version 10.1 of MEGA software (https://www.megasoftware.net/). The 16S rRNA gene sequences of closely related species were retrieved from NCBI GenBank databases. Evolutionary distance was computed using Maximum Composite Likelihood method. Bootstrap values (percentage of 1000 replicates) higher than 50% are shown at node points. Bar, 0.0050 substitutions per nucleotide position.

The microscopic characterization of cells of strain S3 has been revealed by the scanning electron microscopic (SEM) (Fig. S1). Cells are Gram-negative, aerobic, rod-shaped, 1.042–1.236 μm long and 0.6–0.8 μm wide. The strain S3 formed yellowish, circular and smooth colonies on LB agar growth medium after 24 h at 28 °C. Additionally, the phenotypic characterization of isolate S3 was done based on biochemical tests (Table S1). Analysis of fatty acid (FAMEs) profiles of the most promising ACC deaminase producing bacterial strain S3 revealed the presence of major fatty acids peaks, separated by GC with MIDI system (Table S2). The predominant cellular fatty acids in strain S3 was C_16:0_ (26.07%) while C_17:0_ cyclo (6.21%) and C_12:0_ (3.53%) were present in moderate amounts. The isolate showed maximum similarity index (0.556) with *Pseudomonas-putida-biotype A* which was later confirmed by 16S rRNA gene sequencing (Table S3). The phylogenetic characterization of S3 (Accession No. MT860297.1) was done based on the 16S rRNA gene sequence similarity analysis using EzBioCloud server (https://www.ezbiocloud.net) which indicated that strain S3 was related to genus *Pseudomonas* and its closely related species was *Pseudomonas* sp. strain BML3 (Accession No. MK680061.1) (Table S3). The 16S rRNA gene sequences of S3 was deposited in the NCBI GenBank database under the accession number MT860297 as *Pseudomonas* sp. strain S3. The neighbour-joining phylogenetic tree of ACC deaminase producing test strain S3 based on 16S rRNA gene sequence revealed their relatedness with other strains of respective using MEGA X software (Fig. 1).

### Functional characterization of ACC deaminase producing S3 based on PGP traits

**Figure 2:**
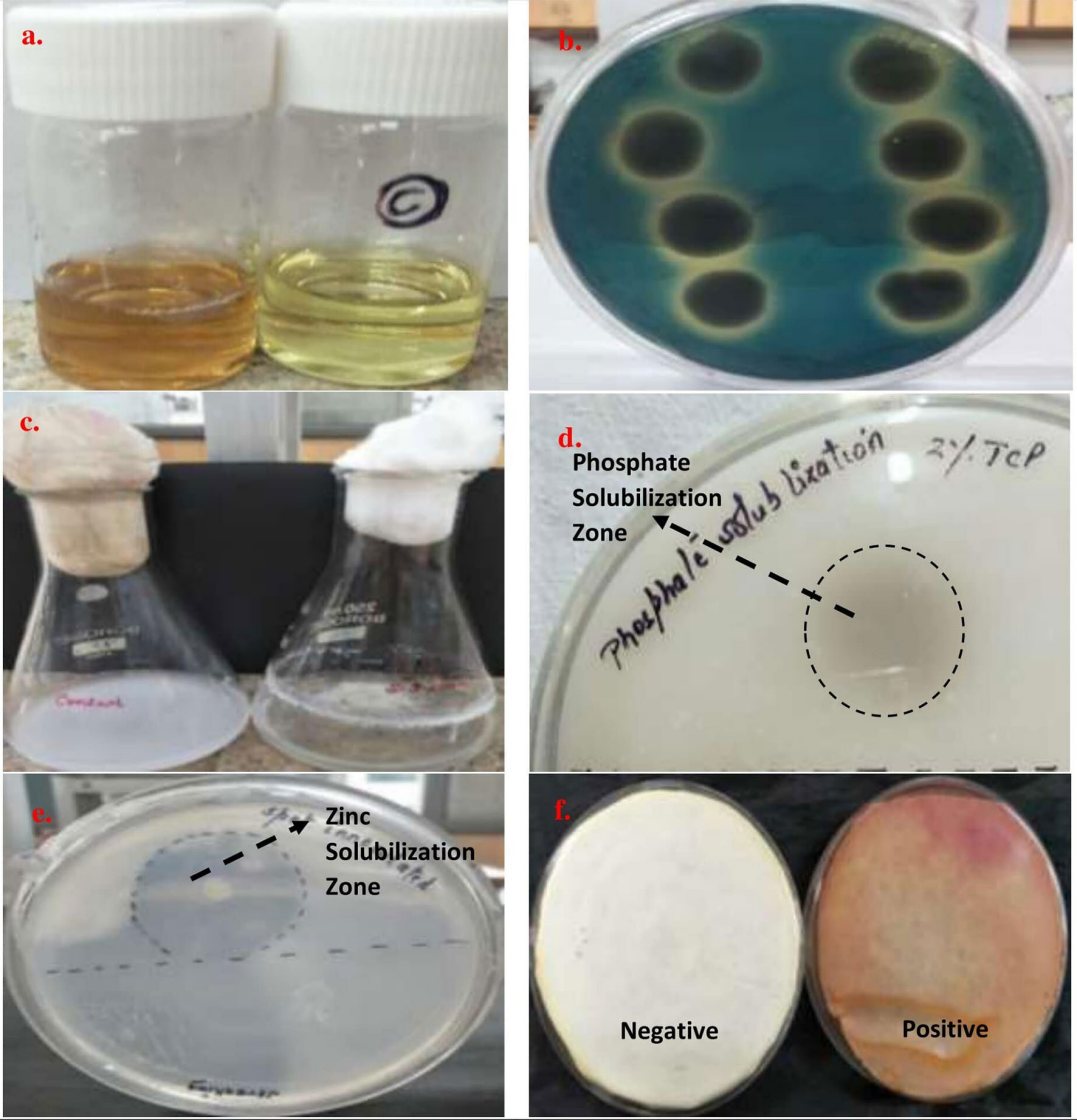
Plant growth promoting analysis of strain *Pseudomonas* sp. strain S3 (**a**) IAA production producing red colour on addition of Salkowski reagent to cell free supernatant; (**b**) Siderophore Production on CAS agar medium; (**c**) Tricalcium Phosphate solubilization in NBRIP medium; (**d**) Tricalcium Phosphate solubilization in Pikovaskya’s agar medium amended with 2% Ca_3_(PO_4_)_2_; (**e**) Zinc Solubilization on Tris minimal medium with 0.1% Zinc oxide; (**f**) HCN production on King’s B medium with red colour of Whatman filter paper saturated with alkaline picric acid solution.

Analysis of multifarious plant growth promoting traits of isolate S3 revealed the positive results for Indoleacetic acid production, Phosphate Solubilization, Siderophore, ammonia and Hydrogen cyanide (HCN) production. The bacterium *Pseudomonas* sp. strain S3 had ACC deaminase activity of 724.56 ± 0.0058 nmol α-ketobutyrate per mg of cellular protein per hour, produced 37.719 μg/mL IAA in the presence of 5 mM tryptophan and solubilized inorganic tricalcium phosphate complex (69.68 mg/L soluble P), visualized by clear halo around the colony on solid Pikovaskya’s agar medium. Additionally, strain S3 solubilize zinc complex, Zinc oxide, showed clear zone around colony spotted and streaked on Tris-minimal medium. The visualization of orange halo around the colony of S3 strain on blue Chrome Azurol S agar medium indicated the production of siderophore. The isolate has also exhibited positive result for hydrogen cyanide production qualitatively (Fig. 2).

### In vitro antagonism assay and scanning electron microscopic (SEM) analysis of biocontrol activity of S3 against *Rhizoctonia solani*

**Figure 3:**
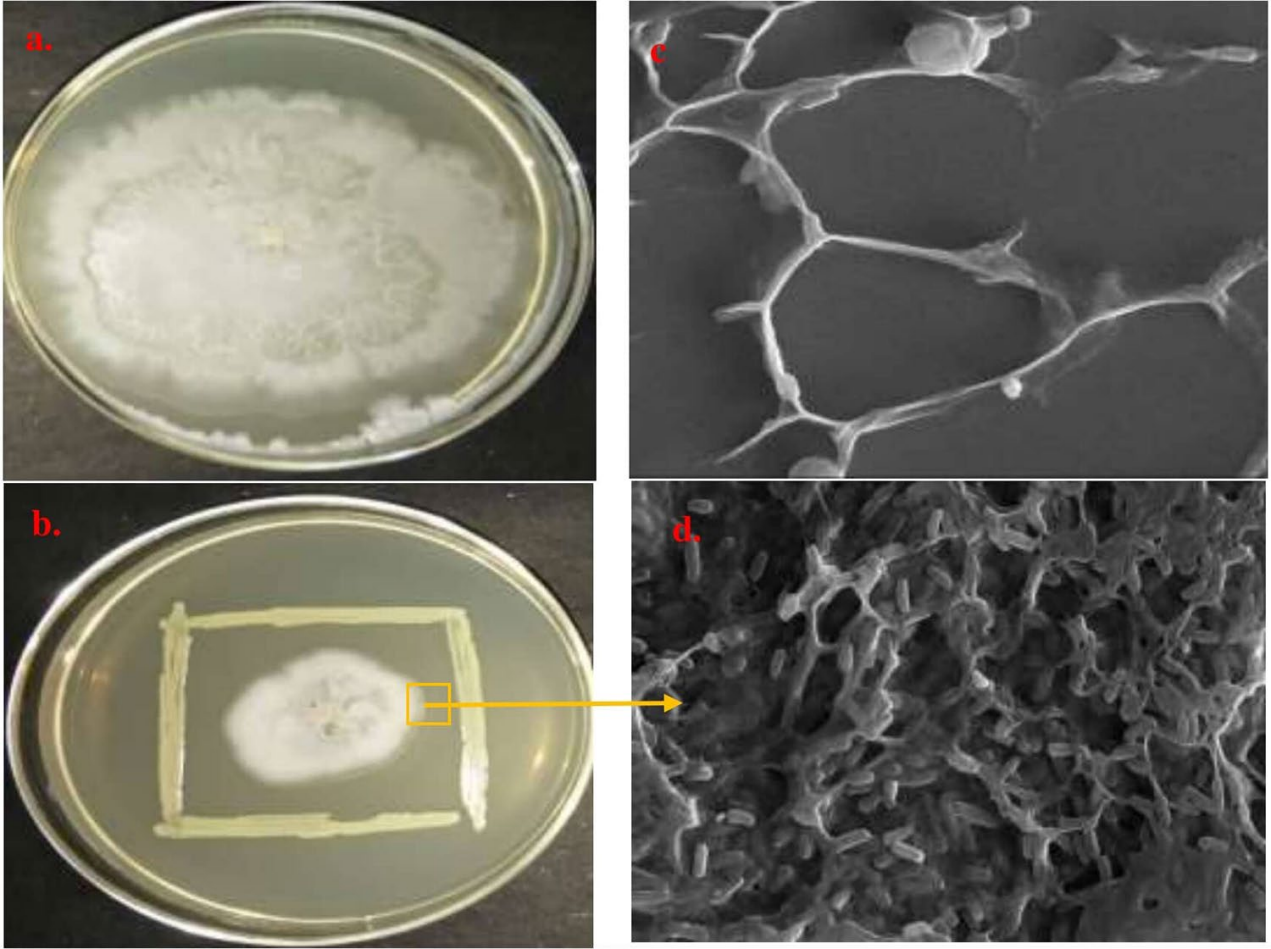
In vitro antagonism of strain S3 against *Rhizoctonia solani* with plates (**a**,**b**) are control of *Rhizoctonia solani* and dual cultures of S3 and fungal pathogen, respectively on Potato Dextrose agar growth medium. While (**c**,**d**) are SEM microphotographs of corresponding pathogenic fungus and antagonistic interaction between strain S3 and *Rhizoctonia solani,* respectively*.* Scale Bar = 2 μm.

The ACC deaminase producing rhizobacterial isolate S3 inhibited the growth of phytopathogen *Rhizoctonia solani* under dual culture assay. The antagonistic interaction between test PGPR *Pseudomonas* sp. strain S3 and fungal pathogen was observed by Scanning electron microscopy (SEM) which clearly showed the adhesion of bacteria to fungus mycelial structure while micrographs of control plate showed intact, undistorted fungus hyphal structures (Fig. 3).

### Determination of bacterial effect on seed germination by greenhouse assay

**Figure 4:**
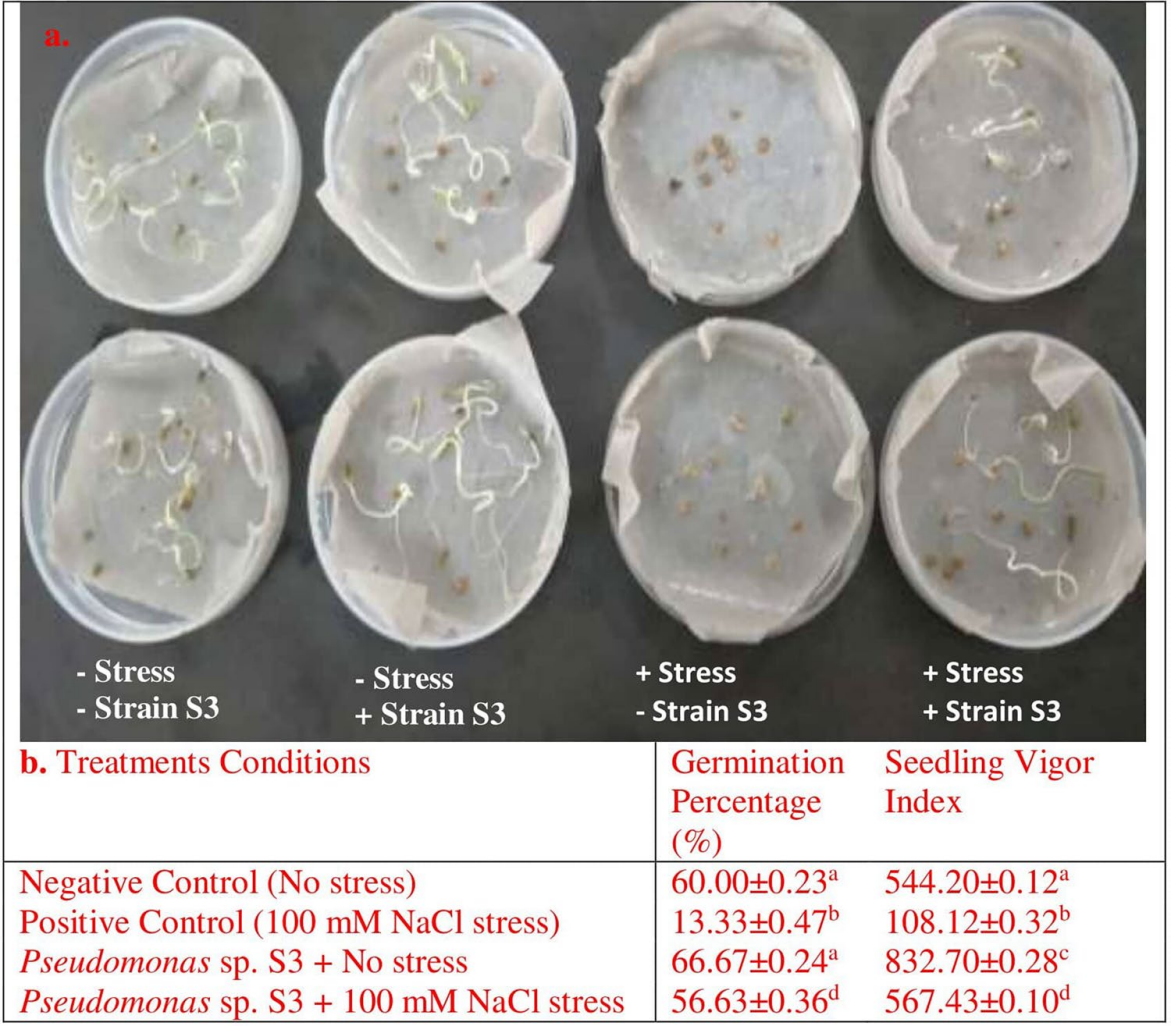
Effect of *Pseudomonas* sp. S3 inoculation and salinity stress (100 mM NaCl) on the germination of tomato (*Solanum lycopersicum*). (**a**) Sterilized uninoculated tomato seeds and S3 strain inoculated tomato seeds in the Petri dishes coupled with filter paper and irrigated with normal water (on the left) and 100 mM NaCl solution (on the right); (**b**) Germination percentage and seedling vigor index of tomato seeds after 10 days of sowing at 28 °C under normal and salinity stress conditions. Values are Mean values ± Standard Deviation from three replicates (n = 3 replicates per treatment). Different letters indicate statistical difference between treatments (Turkey’s Post Test, *P* < *0.05*) under normal and saline conditions.

The tomato seeds bacterized with ACC deaminase producing bacterial strain S3, suspended in 0.03 M MgSO_4_ was investigated for their potential beneficial effects on seed germination under both non-stressed and salt stressed environment (Fig. 4). At normal conditions, no significant influence was observed in germination percentage by bacterial inoculation. The artificial salinization with 100 mM NaCl has significantly decreased the seed germination rate by ~ 78% as compared to untreated seeds (imbibed with only distilled water). However, the bacterial treatment had significant effect on seed germination, resulted in 56.63% seed germination under stressed conditions in comparison to control group. Evaluation of seedling vigor index has shown that *Pseudomonas* sp. S3 has enhanced the vigor index of tomato seedlings (832.70 and 567.43) in comparison to respective control unbacterized groups of seedlings under normal and saline stress conditions.

### Effect of bacterial inoculation on plant growth and biomass under stressed conditions

**Figure 5:**
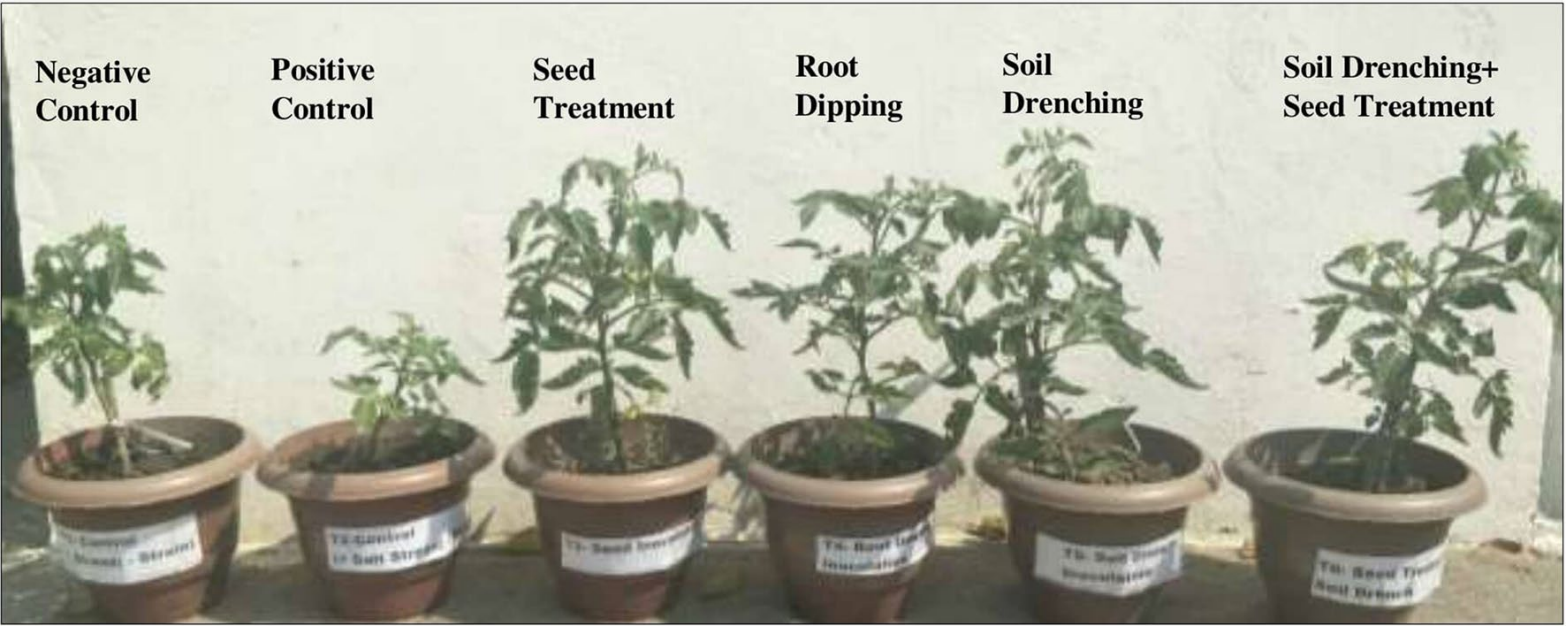
Plant growth promotion in tomato (*Solanum lycopersicum*) plants upon ACC deaminase producing *Pseudomonas* sp. S3 inoculation through employment of different methods—seed treatment, root dipping, soil drenching and combination of seed treatment and soil drenching under non-saline and saline stress conditions.

The effect of bacterial inoculation, method of inoculation and saline environment on plant growth was evaluated by setting pot experiments under open field conditions as shown in Fig. 5.

### Analysis of growth parameter of tomato plants

The plant growth and development were investigated in terms of root fresh and dry weight, shoot fresh and dry weight, root and shoot length as shown in Table 1. The growth parameters were significantly (*P* < *0.05*) enhanced upon bacterial inoculation. Combine method of strain S3 inoculation through seed bacterization and soil drenching resulted in increased biomass production and root and shoot length in comparison to other treatment conditions and control plants in saline stress conditions.

**Table 1:** Effect of different method of bacterial inoculation on root length, shoot length, root fresh and dry weight, shoot fresh and dry weight of tomato plants harvested after 30 days under saline stress and normal conditions.

Treatments	Root system of tomato plants			Shoot system of tomato plants		
	Root Length (cm)	Root fresh Weight (g)	Root dry weight (g)	Shoot Length (cm)	Shoot fresh Weight (g)	Shoot dry Weight (g)
**Normal conditions**						
Negative control	9.1 ± 0.32^a^	2.54 ± 0.04^a^	0.23 ± 0.078^a^	24.8 ± 1.2^a^	10.9 ± 0.96^a^	0.98 ± 0.047^a^
Seed treatment	11.9 ± 0.89	3.7 ± 0.09	0.49 ± 0.035	9.4 ± 0.32	2.38 ± 0.07	0.44 ± 0.067
Root dipping	10.5 ± 0.54	2.8 ± 0.05	0.31 ± 0.085	7.7 ± 0.61	1.85 ± 0.10	0.29 ± 0.033
Soil drenching	13.1 ± 0.74	3.4 ± 0.03	0.57 ± 0.014	12.7 ± 0.96	2.90 ± 0.025	0.50 ± 0.048
Seed treatment + soil drenching	15.0 ± 0.35	4.6 ± 0.08	0.88 ± 0.029	14.3 ± 0.81	3.52 ± 0.098	0.72 ± 0.046
**Saline conditions**						
Positive control	5.8 ± 0.54^b^	1.01 ± 0.24^b^	0.05 ± 0.007^b^	14.2 ± 1.9^b^	4.56 ± 1.25^b^	0.23 ± 0.001^b^
Seed treatment	9.4 ± 0.32^a^	2.38 ± 0.07^a^	0.44 ± 0.067^c^	22.3 ± 1.43^c^	8.75 ± 1.26^c^	1.85 ± 0.047^c^
Root dipping	7.7 ± 0.61^c^	1.85 ± 0.10^c^	0.29 ± 0.033^a^	20.2 ± 0.94^d^	10.09 ± 0.73^d^	0.58 ± 0.041^d^
Soil drenching	12.7 ± 0.96^d^	2.90 ± 0.025^a^	0.50 ± 0.048^c^	24.0 ± 0.86^a^	13.43 ± 1.22^e^	1.94 ± 0.058^c^
Seed treatment + soil drenching	14.3 ± 0.81^e^	3.52 ± 0.098^d^	0.72 ± 0.046^d^	32.2 ± 1.03^e^	15.78 ± 1.16f.	2.80 ± 0.018^e^

Values are Mean values ± Standard Deviation from three replicates (n = 3 replicates per treatment). Different letters indicate statistical difference between treatments (Turkey’s Post Test, *P* < *0.05*) under normal and saline conditions.

### Analysis of stress ethylene formation

**Figure 6:**
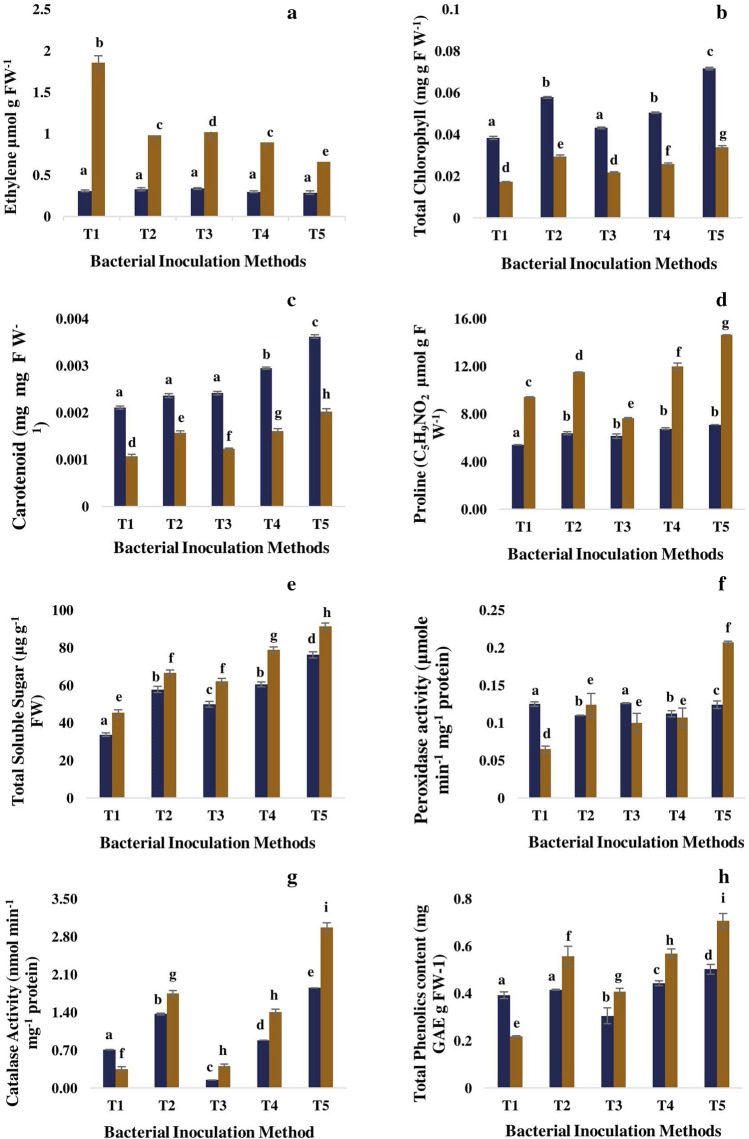
Effect of ACC deaminase producing S3 and different inoculation method on (**a**) Ethylene production, (**b**, **d**) photosynthesis related parameters, (**d**, **e**) osmoprotectants production, (**f**, **g**) antioxidants levels, (**h**) phenolic content of tomato plants of respective treatments. Columns represent Mean values ± standard deviation (n = 3 replicates per treatment). T1: control (positive and negative control); T2: seed treatment; T3: root dipping; T4: soil drenching; T5: seed treatment + soil drench; indigo bar, normal conditions; brown bar, saline conditions; different letters indicate statistical difference between treatments (Turkey’s post test, *P* < *0.05*) under normal and saline conditions.

The ethylene production was increased in tomato plants by 5.00-fold in response to 100 mM NaCl stress conditions as compared to untreated non-stressed plants. But, the priming of seeds with ACC deaminase producing inoculant S3 had reduced levels of 100 mM NaCl salt stress-induced ethylene and thereby improved biomass production and growth of salt-stressed plants (Fig. 6). A significant reduction (61%, *P* < *0.05*) was observed in Treatment 5 consisting of seed priming and soil drenching method of inoculation followed by Soil drenching (50%) and Seed bacterization (46%), individual method of inoculation in comparison to uninoculated control.

### Effect on leaf photosynthetic pigments: total chlorophyll and carotenoid content

The influence of different inoculation methods of bacterial strain S3 and salinity stress on leaf photosynthetic pigments, total chlorophyll and carotenoids are illustrated in Fig. 6. Under normal conditions, application of ACC deaminase producing PGPR strain S3, regardless of any inoculation method, has improved the production of chlorophyll (12–86%) and carotenoids (11–71%) in comparison to positive control. On exposure to salt (100 mM NaCl) stress conditions leaves became chlorotic and yellowish in colour, indicated significant reduction in total chlorophyll by 55% (*P* < *0.05*), and carotenoid by 49% (*P* < *0.05*) which leads to impairment of photosynthetic machinery of salt-stressed plants in comparison to positive control. The combination of seed inoculation and soil drenching has resulted in the maximum production of chlorophyll (0.034) and carotenoids (0.0020) content while root dipping inoculation method yielded compartively lower levels of these pigments in salt stress tomato plants.

### Effect on osmolytes accumulation: Proline and total soluble sugar content

The ACC deaminase producing rhizobacterial strain S3 was further investigated for the ability to confer osmotolerance to maintain osmotic potential in Tomato plants as shown in Fig. 6.

Under non-saline conditions, the inoculation of tomato plants with *Pseudomonas* sp. strain S3 has enhanced the accumulation of proline but no significant difference (*P* < *0.05*) was found among four different delivery methods. We observed that the accumulation of proline was increased in all inoculated (~ 24–106%) and non-inoculated plants (~ 70%) under salinity stress conditions as compared to corresponding non-saline plants.

The biosynthesis of total soluble sugar in tomato plants under salinity stress induced by 100 mM NaCl was increased by 34% with respect to untreated non-stressed plants. The plants inoculated with *Pseudomonas* sp. strain S3 have significantly (*P* < *0.05*) enhanced the total soluble sugar content of plants in the range of 0.37–1.01-fold in comparison to negative control plants. The introduction of bacterial strain S3 through combination of seed inoculation and soil drenching method has recorded the highest accumulation of proline and total soluble sugar by ~ 55% and 101%, respectively while the minimum was found with root dipping method in comparison to control uninoculated plants.

Therefore, the ACC deaminase producing strain S3 was effective when seeds were imbibed in bacterial suspension together with soil drenching method to alleviate the osmotic stress by inducing accumulation of osmolytes like soluble sugar and proline in plants and thus, improved the plant growth under salt stressed conditions.

### Effect on leaf enzymatic antioxidants and phenolics content analysis

The efficacy of ACC utilizing bacterial strain S3 to stimulate the plant’s inherent antioxidative defensive system and phenolics content under salinity stress was also determined (Fig. 6).

Under normal conditions the levels of catalase and peroxidase antioxidative enzymes were similar in all groups of uninoculated and bacterial inoculated plants. The uninoculated plants subjected to 100 mM salinity stress has shown the decreased antioxidants activity such as peroxidase (POD) and Catalase (CAT) by 48% and 50%, respectively in comparison to non-inoculated normally watered plants. However, tomato plants treated with bacterial strain S3, have significantly exhibited higher antioxidative levels of POD and CAT activity, irrespective of inoculation method, when exposed to salt stress. However, plants bacterized through seed inoculation and soil drenching method recorder significantly higher enzymatic content of POD and CAT by 2.18-fold and 7.51-fold, respectively in comparison to control plants.

Under normal conditions, bacterial inoculation has increased the phenolics content in tomato plants in relation to that of non-inoculated, however, no marked difference was reported among the bacterial treatment methods. The 100 mM NaCl salt stress has reduced the phenolic content, the beneficial Phyto antioxidants by 44% in comparison to normally watered plants. The phenolics content of leaves of salt exposed tomato plants was increased significantly, in the presence of ACC deaminase inoculant S3. Furthermore, the bacterized plants through seed treatment and soil drenching method have a higher content of plant phenolics compounds by ~ 200% as compared to negative control group (Fig. 6).

## Discussion

The application of beneficial rhizobacteria for plant growth promotion and maintenance of soil health is widely reported. This approach is considered as a sustainable technology that will fulfil the requirements of the increasing population without compromising the soil, plant, or human health^18–20^. Employment of ACC deaminase producing rhizobacterial strains is one of the futuristic approaches substituting the use of agrochemicals for enhancement of agricultural yield under salinity stressed conditions by optimizing the levels of stress-ethylene in salt exposed plants^21,22^.

Therefore, the present study was designed to isolate ACC deaminase producing bacteria from rhizospheric soil of turmeric (*Curcuma longa*) growing organically in Motihari district of Bihar, India. A total of 10 bacterial isolates were obtained, out of which one was able to grow on DF minimal salt medium utilizing ACC (1-aminocyclopropane-1-carboxylate) as the nitrogen source. Bacteria producing ACC deaminase in the rhizosphere of crops are known to alleviate various biotic and abiotic stresses in plants^23–25^. In the present study, apart from ACC deaminase activity, other direct PGP traits like production of Indoleacetic acid (auxin), Fe-chelating siderophore, Hydrocyanic acid, solubilization of insoluble inorganic phosphate and zinc sources, ammonia production as well as antagonistic activity against soil borne fungal pathogen were also evaluated.

There are several reports suggesting the application of plant growth promoting bacteria with multifarious PGP traits improve the plant biomass and yield by suppressing the effect of biotic and abiotic stressed conditions on plants^26,27^. It was found that bacterial inoculation significantly enhanced the germination percentage under 100 mM NaCl stress condition. Our results are in agreement with published literature who have reported that PGPR could alleviate the detrimental effect of salinity on germination^28–30^. The production of the stress hormone ethylene has been reported in various crop plants growing under stressful conditions which is attributed to inhibition of root development, root nodulation in leguminous crops, senescence and abscission process in leaves, impairment of chlorophyll and its associated photosynthetic apparatus^31^. PGPR with ACC deaminase activity having the potentiality to reduce ethylene concentration is one of the most effective and alternative strategies to induce tolerance in plants against various abiotic and biotic stressed conditions^22,32^. To date, several studies have been carried out, in which PGPR was utilized for their potent role in salinity stress mitigation^33–37^. *Pseudomonas* sp. strain S3 found in this study was able to control the stress induced by salinity which is in line with previous reports that demonstrated the potentiality of PGPR belonging to genus *Pseudomonas* to impart salt tolerance by the production of plant growth regulators such as indoleacetic acid as well as iron-chelating siderophores, solubilization of insoluble complexes of nutrient including phosphate and zinc and thus, improving plant biomass under salt-stressed conditions^38–40^ .

*Rhizoctonia solani* is an important soil-borne plant pathogen causing root rot disease in various crops causing significant agricultural losses. In this work, apart from plant growth promoting potential, *Pseudomonas* sp. strain S3 was characterized as potential biocontrol agent against phytopathogenic fungi-*Rhizoctonia solani* which is in agreement with previous work reporting the broad spectrum of antifungal activity of different species of genus *Pseudomonas* against pathogenic fungi such as *Fusarium oxysporum*, *Rhizoctonia solani*, *Botrytis cinerea, Alternaria alternata, Aspergillus niger*^42–45^.

The effect of inoculation of *Pseudomonas* sp. strain S3 in stimulating the growth and development of tomato plants under saline stressed conditions was carried out using pot trials under field conditions. The results showed plant growth parameters of tomato plants such as root length, shoot length, plant dry and fresh biomass (roots and shoots biomass) were enhanced significantly by inoculation with ACC deaminase exhibiting *Pseudomonas* sp. strain S3 as compared to non-inoculated plants under salt stress which is in line with that of Chatterjee et al.^46^. Also, bacterial inoculation with strain S3 had considerable influence in enhancing the seed germination rate of tomato plants in saline conditions. Inoculation of ACC degrading bacterial strains decreased ethylene levels by irreversibly breakdown stress induced ACC into α-ketobutyrate and ammonia instead of ethylene and therefore alleviates its potentially toxic effects manifested on plants in response to stress^47^. These findings are parallel to some previous research studies in which it was demonstrated that bacterial strain expressing ACC deaminase activity counteracts the salt stress induced growth inhibition primarily through regulating the ethylene synthesis in various crop plants^41,48–50^. The inoculation of tomato plants with ACC deaminase producing bacterial strain S3 has significantly enhanced the total chlorophyll content, carotenoid content and therefore improve the photosynthetic activity of salt-stressed tomato plants which is in line with that of Yasmin et al*.*^51^. Moreover, the accumulation of low molecular weight, compatible solutes are also known as osmoregulators such as soluble sugars, proline, trehalose, glycine betaine, etc. was induced in untreated salt stressed tomato plants to maintain water balance and withstand the salinity stressed conditions^52^. The results suggested that *Pseudomonas* sp. strain S3 has facilitated growth and allowed tomato seedlings to thrive under salinity stress conditions by accumulating more proline and soluble sugars content in comparison to uninoculated plants. The findings are incongruence with other findings in which the ACC deaminase producing *Bacillus* sp. mitigated the adverse effects of salt stress on the osmotic balance of plants^53,54^. The salinity stress has drastically induced autonomous oxidative stress in plants mediated by inefficient detoxification by the defensive system including enzymatic (superoxide dismutase, catalase, peroxidase, and polyphenol oxidase) antioxidants. The activities of catalase and peroxidase were decreased in salt stressed plants while inoculation with bacterium *Pseudomonas* sp. strain S3 has enhanced the activities of these enzymes under salinity stress conditions. The observed results were in agreement with Karthikeyan et al*.*^55^ which demonstrated that ACC deaminase producing bacterial strain *Achromobacter xylosoxidans* has mitigated the salinity effects by modulating the functions of enzymatic antioxidants of *Catharanthus roseus* grown in saline soil. Win et al*.*^56^ showed ACC deaminase producing *Pseudomonas* spp. conferring salinity tolerance in tomato plants by stimulating the defensive activity of antioxidants. Besides, the phenolic content was also enhanced in leaves of tomato plants inoculated with *Pseudomonas* sp. strain S3 to counter the effects of oxidative stress induced in response to salinity stress. This is in line with earlier studies which determined the efficiency of *Pseudomonas* species in enhancing total phenolics content in crops plants for instance, soybean, finger millet, *Mentha piperita* grown under stressed conditions such as drought and salinity^57–59^.

For application of bacterial inoculant, four different delivery method were employed including seed treatment, soil drenching root dipping and soil drenching + seed treatment method. In most of the studies, seed coating or bacterization was found to be most commonly used and well-established practice^60^, however in the present study, this method of delivery was found to be less effective. The application of bacteria through seed bio-priming along with soil drenching has significantly enhanced plant growth as compared to other bacterial application mode through seed inoculation, soil drenching and root dipping methods. The inoculation of tomato plants with *Pseudomonas* sp. strain S3 by the combination of seed treatment and soil drench has recorded significantly increased morphological, physiological and biochemical attributes which is considered responsible for salinity stress tolerance mechanism.

In conclusion, this study revealed the plant growth promoting potential of rhizospheric turmeric isolate *Pseudomonas* sp. S3 in tomato plants grown under salinity stress conditions. Moreover, the results indicated that different inoculation methods influence the stress tolerance and plant growth promoting efficiency of bacterial strain. The inoculation of tomato plants though the combination of seed bacterization and soil drenching method resulted in higher germination rates, seedling vigour index, increased length, fresh and dry weight of root/shoot biomass, enhanced photosynthetic pigment content, and stimulated the production of antioxidants such as POD, CAT activities, phenolic content as well as osmoprotectants content of tomato plants. Besides, ACC deaminase activity for reduction of salt stress induced ethylene, the other direct inherent PGP traits of *Pseudomonas* sp. S3 such as phosphate solubilization, zinc solubilization, IAA production, siderophore, ammonia and HCN production might be responsible for alleviation of salt stress in tomato plants. Further evaluation with respect to actual field trials will be required to confirmed the effective utility of ACC rhizospheric bacterial strain, *Pseudomonas* sp. S3 as bio-inoculant for mitigation of negative effects of salt stress on tomato plants.

## Methods

### Isolation of rhizobacteria, preliminary screening and quantification of ACC (1-aminocyclopropane-1-carboxylate) deaminase activity

The rhizobacteria were enumerated from soil samples of *Curcuma longa* (Turmeric), member of *Zingiberaceae* family, collected from Motihari district of Bihar, India by using serial dilution of soil suspension on Luria–Bertani agar medium.

The morphologically different 10 bacterial colonies were randomly picked and re-streaked on sterile minimal DF (Dworkin and Foster) salt media^60^ amended with 3 mM ACC (Sigma-Aldrich Inc. MO, USA) instead of (NH_4_)_2_SO_4_ as sole nitrogen source. The plates were incubated for three days at 28 °C and observed for growth and appearance of bacterial colonies on all minimal agar plates. The selected isolate S3 was taken as ACC deaminase producer and was purified by sub culturing the isolate.

The ACC deaminase activity of the selected isolate was determined quantitively in terms of α-ketobutyrate produced during hydrolysis of ACC. The cell pellet from the overnight grown culture of selected ACC deaminase producing isolate in DF-ACC minimal medium was collected by centrifugation at 8000 g for 10 min. The pelleted cells were washed repeatedly two–three times with 0.1 M Tris–HCl (pH 7.6) and centrifuged at 16,000 g for 5 min. The washed pelleted cells were again suspended in 0.1 M Tris–HCl (pH 8.5) and treated with 30 µl toluene as well as with 20 µl 0.5 M ACC. The resultant cell suspension was briefly vortexed and incubated for 15 min at 30 °C. Thereupon, one mL of 0.56 M HCl and 300 µl 2, 4-dinitrophenylhydrazine reagent was added to 1 mL of ACC treated tolunized cell suspension. Lastly, 2 mL of 2 N NaOH was added to the final reaction mixture and optical density of the mixture was measured at 540 nm in a UV–Vis spectrophotometer. The ACC deaminase was measured by comparing with the standard curve of α-ketobutyrate (Sigma-Aldrich Inc. MO, USA) ranged from 0.1 to 1.0 µmol and expressed as nmol α-ketobutyrate mg^−1^ protein hr^−1^^61^. The protein estimation was done as per Bradford methodology using BSA (Bovine Serum Albumin) as standard^62^.

### SEM Imaging of ACC deaminase producing bacteria

The general morphology of bacterial strain S3 was observed by routinely cultivating on DF-ACC agar medium at 28 °C for 24 h and furthermore, investigated by ZEISS EVO scanning electron microscope at Amity Institute of Renewable and Alternative Energy, Instrumentation facility, Noida, Uttar Pradesh, India. The bacterial strain was inoculated in LB broth medium for overnight incubation and then harvested by centrifugation at 2000 g for 5 min. The resultant cell pellet was washed twice with 0.05 M phosphate buffer (pH 7.3) and fixed with 1.5% glutaraldehyde at 4 °C for 24 h and subsequently, washed thrice with phosphate buffer solution. The cells were then dehydrated with graded series of ethanol (30–100%) at 15 min interval. Followed by drying to remove excess liquid, the dehydrated cells were mounted on a SEM stubs, coated with a layer of gold: palladium (60:40) and imaged using ZEISS EVO SEM and SEM micrographs were recorded.

### Biochemical and fatty acid methyl ester (FAME) characterization

The gram staining was conducted as per Beveridge^63^. Catalase and oxidase assays were conducted by using 3% (v/v) hydrogen peroxide solution and 1% (w/v) tetramethyl-p-phenylenediamine, respectively. While the other conventional biochemical tests such Indole, Methyl-Red, Voges-Proskauer, Citrate Utilization, Carbohydrate Utilization, Urease, Gelatinase production assays were performed and characterized based on Bergy’s manual of Systematic Bacteriology^64^.

The methyl ester of fatty acids of isolate S3 was analysed using automated gas chromatography-based MIDI Sherlock Microbial Identification System. The FAMEs were extracted from ~ 20 mg bacterial cells with liquid–liquid extraction procedure by organic reagent hexane: methyl tert-butyl ether for GC (Agilent Technologies, USA) analysis with Ultra 2 column (25 m × 0.2 mm phenyl methyl silicone fused silica capillary column) and flame ionization detector. The FAMEs were then analysed by the Aerobic library (RTBSA6.0) and samples with a similarity index (SI) of 0.500 or higher were considered as identified.

### PCR amplification and 16S rRNA gene sequencing analysis

The genomic DNA of ACC deaminase producing rhizobacterial strain S3 was isolated according to conventional phenol/chloroform method and used for amplification of 16S rRNA gene encoding region (~ 1500 bp) using two universal 16S rRNA gene primers pA (5′-AGAGTTTGATCCTGGCTCAG-3′) and pH (5′-AAGGAGGTGATCCAGCCGCA-3′) by PCR^65^. A similarity search was performed to compare the resultant sequence with those of other strains in the EzBioCloud database (http://www.ezbiocloud.net/eztaxon) which identify its closest relative and calculate the pairwise sequence similarities. Further, the query sequence and that of related species were aligned using multiple sequence alignment tool CLUSTAL *W* tool in MEGA X program. Phylogenetic tree was constructed by Neighbour-joining (NJ) method using software MEGA X with the bootstrap of 1000 replicates and evolutionary distances were computed using the Maximum Composite Likelihood method^66^.

### Bacterial salt tolerance and functional characterization based on PGP traits

The tolerance of test isolate to salinity stress was studied by visualizing its growth on the Luria–Bertani (LB) agar medium supplemented with 2–8% (w/v) NaCl concentration for 72 h at 28 °C.

The production of IAA was analysed spectrophotometrically at 530 nm with the help of Salkowski reagent. It was visualized by development of pink colour and quantified with the help of standard curve of pure IAA (Hi Media Chemicals, Mumbai, India) obtained in the range of 5–50 µg/ml^67^.

The phosphate solubilization was conducted by spot inoculation of test isolate on Pikovaskya’s agar plates supplemented with 2% (w/v) insoluble inorganic Tricalcium phosphate (Ca_3_(PO_4_)_2_, Hi Media Chemicals, Mumbai, India). Furthermore, the quantitative analysis of Ca_3_(PO_4_)_2_ solubilization was done in National Botanical Research Institute’s Phosphate Growth (NBRIP) medium to estimate the amount of soluble phosphate (Soluble P) as mg/L^68^.

The test isolate was spot inoculated on Tris-minimal medium supplemented with 0.1% (w/v) insoluble Zinc Oxide and monitored for zinc solubilization by formation of halo zones around the colonies after incubation for 14 days at 30 °C^69^.

The selected bacterial isolate was subcultured on King’s B medium supplemented with 0.4% (w/v) glycine for estimation of Hydrogen cyanide (HCN) production. The transformation of yellow alkaline sodium picrate Whatman filter paper into red brown colour indicates the formation of HCN^70^. Estimation of ammonia was carried out by addition of Nessler’s reagent to bacterial culture in peptone water and observed for development of slight yellow to brownish colour^71^.

The development of orange-yellow halo around the bacterial colonies on Chrome Azurol S (CAS) agar plates after incubation at 28 °C. for 4 days, was indicator for siderophore producing bacterial isolates^72^.

### In vitro antifungal activity against *Rhizoctonia solani* and visualization by SEM

The antifungal potential of strain S3 was evaluated against plant pathogen, *Rhizoctonia solani* obtained from Indian Agricultural Research Institute (IARI), Pusa, New Delhi, India by performing dual culture assay. The test isolate was streaked as straight line on four edges around the mycelial disc of fungal pathogen at the centre of Potato dextrose agar medium (PDA, Hi Media Chemicals, Mumbai, India) and incubated for 5 days at 28 °C.

The hyphal area of ~ 4 mm from the bacteria-fungus interaction zone (as shown in Fig. 4) was taken and placed on the glass cover slips and overnight fixation was done in 1.5% glutaraldehyde in 0.05 M phosphate buffer (pH 7.3). Similar procedure was done with fungal mycelium taken from the petri plate grown without bacterial inoculation (control). All the fixed samples were subsequently washed three times with phosphate buffer for 10 min each to remove loosely adhered cells. Followed by dehydration of samples using increasing concentration of ethanol—30–100% ethanol (v/v) at 10 min interval. Finally, the dehydrated samples were mounted on SEM stubs using carbon tapes and coated with gold: palladium (60:40) for visualizing under ZEISS EVO scanning electron microscope and photomicrographs were recorded.

### Effect of bacterial inoculation on tomato (*Solanum lycopersicum*) germination under salinity stress

The bacterial cells were harvested after 3 days incubation in DF-ACC minimal medium in order to induce ACC deaminase activity and then suspended in 0.03 M MgSO_4_ to achieve the requisite concentration of cells at OD_600_ = 1.00 (10^8^ cfu/ml).

The tomato seeds procured from Indian Agricultural Research Institute (IARI), Pusa, New Delhi, India, were used for growth promoting experiments, were surface sterilized by utilizing 70% (v/v) ethanol and 1% (v/v) sodium hypochlorite solution (NaClO). The germination test was performed by placing 10 bacterized and uninoculated seeds on Whatman filter paper in 10 cm Petri dishes (3 replicates per treatment) and incubated for 10 days at 20 °C in the growth chamber. The paper was moistened with normal tap water (control) and saline (100 mM NaCl) solution to artificially induced salinity stress conditions. The Petri dishes with uninoculated seeds exposed to saline and non-saline environment served as negative and positive control respectively^21^.

### Experimental design and treatment for plant growth promotion assay

The growth of Tomato (*Solanum lycopersicum*) was evaluated through pot study under the influence of four delivery methods for inoculating strain S3 and salt (100 mM NaCl) stress conditions. The experiment comprises control group (unbacterized) and four different bacterial inoculation methods—Seed treatment, Root Dipping, Soil Drenching and Combination of Seed treatment and Soil Drenching under non-saline and saline (100 mM NaCl) stress conditions. Each treatment was replicated five times and arranged in completely randomized block design.

The experiment was conducted in 12″ inch pots filled with a sterilized potting mixture of field soil and coco-peat in 1:1 ratio (7 kg soil pot^−1^) in open field conditions at Amity Institute of Organic Agriculture, Amity University, Noida, Uttar Pradesh, India.

In seed treatment method, the surface sterilized tomato seeds were treated with S3 bacterial suspension in 0.03 M MgSO_4_ (10^8^ cfu/ml) for 1 h and sown after air dried aseptically in laminar air flow. While in root dipping method, the roots of tomato seedlings were completely immersed in S3 culture for 30 min prior to transplantation and in soil drenching method, the bacterial inoculation was done by applying 50 mL S3 culture suspension (10^8^ cfu ml^−1^) in the surrounding soil after sowing of tomato seeds. The seeds treated with 0.03 M MgSO_4_ solution without any bacterial inoculation was served as control groups. The uninoculated plants exposed to salinity stress were served as positive control while uninoculated plants grown under normal (non-saline) conditions were represented as negative control.

The inoculated and uninoculated tomato seeds of corresponding treatment were sown at an average of 4 seeds per pot. Five days after emergence of seedlings the pots were thinned to one seedling.

The plants were grown organically without the application of chemical-based fertilizers. The pots were watered regularly, twice a day, with either normal distilled water or saline solution of EC 10 ds m^−1^ (100 mM NaCl) as per the treatment requirement.

### Analysis of morphological growth parameters of tomato plants

Following the bacterial inoculation and salt stress exposure for 30 days the plants were harvested and growth parameters such as root and shoot length, fresh weight of roots and shoots were determined. Furthermore, the roots and shoots of tomato plants were excised with sterile blade and were weighed separately on electronic balance for computing fresh weight. They were also oven dried separately at 60 °C for 3 days till constant weight was attained to calculate the dry biomass of plants^73^.

### Analysis of ethylene emission and total phenolic contents

The ethylene production was assessed by tomato seedling placed inside the 60-mL glass tubes and sealed with rubber septum for 4 days at 28 °C. The amount of ethylene present in 1-mL of headspace gas was detected by Gas Chromatograph (Bruker 450-GC, Bruker Corporation, United States) equipped with flame ionization detector (FID) and expressed as μM ethylene g^−1^ of Fresh weight (FW) by comparing with the standard curve of pure ethylene^41^.

The total phenolic content was measured at 750 nm according to Siddiqui et al.^74^ using Folin Ciocalteu reagent and Gallic acid as standard. The phenolic content in ethanolic leaf extract mixture was expressed in terms of Gallic acid equivalents (GAE) as mg GAE per mg Fresh weight (FW).

### Determination of photosynthetic pigments: chlorophyll and carotenoid

The total leaf chlorophyll content was determined according to Hiscox and Israelstam^75^and calculated as mg g^−1^ of Fresh weight (FW) as per Arnon^76^. Similarly, total carotenoid content was determined using the formula of Sarkar and Oba^77^.

### Analysis of total proline and soluble sugars content of leaves

The proline content in fresh leaves of tomato plants was measured by spectrophotometric absorption at 520 nm following Bates et al.^78^. The proline concentration was quantified by using the standard curve of L-proline and expressed as μmol g^−1^ fresh weight.

The measurement of total soluble carbohydrate content was performed in both fresh normal and saline water treated plant material using the phenol sulfuric reagent method at absorbance 490 nm as per Dubios et al.^79^.

### Determination of antioxidant enzymatic activities of plants under normal and salt-stressed conditions

The representative leaves samples (1 g) from each treatment were macerated with homogenizing buffer of 50 mM cold phosphate buffer (pH 7.2) in pre-chilled mortar and pestle. The homogenate was centrifuged at 15,000 rpm for 15 min at 4 °C and supernatant was used as crude extract for protein estimation and for antioxidant enzyme-Catalase (CAT), Peroxidase (POD) activities^80^.

Protein concentration in tomato plants in both normal and salt-stressed conditions was determined according to the Lowry method of protein estimation using Bovine serum albumin (BSA) as standard^81^.

The activity of catalase enzyme (CAT; 1.11.1.6) was determined by the degradation of H_2_O_2_ as per Beer and Sizer^82^. The amount of catalase was calculated by the molar extinction coefficient of H_2_O_2_ (36 M^−1^ cm^−1^) and expressed as nmol H_2_O_2_ min^−1^ mg^−1^ protein.

The activity of Peroxidase (POD; EC 1.11.1.x) was measured by monitoring the increase in absorbance at 420 nm at regular interval of 20 s for 3 min using pyrogallol as a substrate and calculated as enzyme unit per mg protein^83^. One unit of peroxidase enzyme was defined as amount of enzyme required to form 1milligram of purpurogallin in 20 s.

### Statistical analysis

All data of tomato growth parameters obtained from plant growth promotion assay were analysed by one-way ANOVA followed by Tukey’s test with bacterial inoculation method considered as independent variable. All the statistical analyses were calculated at significance level *p* = *0.05* through SPSS software. The experiments were performed in triplicates, the mean and standard deviation were calculated using Microsoft Excel 2016.

## Supplementary information

Supplementary Information 1.
