# Supplementary material for: Evaluation of Pseudomonas sp. for its multifarious plant growth promoting potential and its ability to alleviate biotic and abiotic stress in tomato (Solanum lycopersicum) plants
Source: Sci Rep. 2020 Dec 1;10:20951. doi: 10.1038/s41598-020-77850-0 (PMC7708495; doi:10.1038/s41598-020-77850-0)
Supplement: Supplementary file 1 — Supplementary Information 1. [file 41598_2020_77850_MOESM1_ESM.docx]

**Evaluation of *Pseudomonas* sp. for its multifarious plant growth promoting potential and its ability to alleviate biotic and abiotic stress in tomato (*Solanum lycopersicum*) plants**

Sangeeta Pandey* and Shikha Gupta

Amity Institute of Organic Agriculture, Amity University Uttar Pradesh, Sector 125, Noida, Uttar Pradesh, India. 201313

Corresponding author

Dr. Sangeeta Pandey

Email id; [spandey5@amity.edu](mailto:spandey5@amity.edu), sangeetamicro@gmail.com

Amity institute of Organic Agriculture

Amity University Uttar Pradesh

Sector 125, Noida,

Uttar Pradesh 201313, India

**Supplementary Figures**

**Figure S1**. The photograph of ACC deaminase producing *Pseudomonas* sp. S3 colony grown on DF agar minimal plates at 28℃ for 48 hours (a); Scanning electron micrographs displaying rod shaped morphology of *Pseudomonas* sp. S3. Scale Bar = 200 nm.

**Supplementary Tables**

**Table S1.** Biochemical characterization of ACC deaminase producing bacterial isolate *Pseudomonas* sp. strain S3

**Table S2.** Fatty acid profile study of ACC utilizing *Pseudomonas* sp. strain S3 using GC-MIDI Sherlock Microbial Identification System.

**Table S3.** Identification of root associated, ACC deaminase producing *Pseudomonas* sp. strain S3 by means of fatty acid methyl ester (FAME)Analysis and partial sequencing of 16S rRNA gene

| 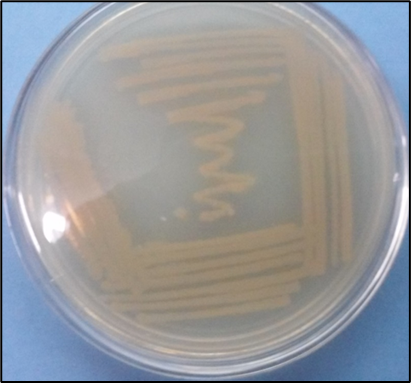  **S3 strain**  **a.** |   **b.** |
| --- | --- |

**Figure S1.** The photograph of ACC deaminase producing *Pseudomonas* sp. S3 colony grown on DF agar minimal plates at 28℃ for 48 hours (a); Scanning electron micrographs displaying rod shaped morphology of *Pseudomonas* sp. S3. Scale Bar = 200 nm.

**Table S1.** Biochemical characterization of ACC deaminase producing bacterial isolate *Pseudomonas* sp. strain S3

| Biochemical traits | Indole | MR | VP | CIT | GEL | CAT | OXID | LAC | SUC | DEX | Identification |
| --- | --- | --- | --- | --- | --- | --- | --- | --- | --- | --- | --- |
| S3 | + | + | - | + | - | + | + | + | + | - | *Pseudomonas* spp. |

Symbols: +, positive; -, negative. Indole, Indole test; MR, Methyl Red Test; VP, Voges-Proskauer; GEL, Gelatin Hydrolysis; CAT, Catalase; OXID, Oxidase test; LAC, Lactose Fermentation; SUC, Sucrose Fermentation; DEX, Dextrose Fermentation; CIT, Citrate Utilization. Positive for Indole production, methyl-red test, citrate utilization, catalase and oxidase production, negative for Voges-Proskauer test, Gelatin hydrolysis. Strain S3 ferment Lactose and Sucrose while No fermentation of Dextrose.

**Table S2.** Fatty acid profile study of ACC utilizing *Pseudomonas* sp. strain S3 using GC-MIDI Sherlock Microbial Identification System.

| Name of Fatty acids | Percentage of fatty acid |
| --- | --- |
| Straight Chain Fatty acid | |
| C_10:0_ | 0.48 |
| **C_12:0_** | **3.53** |
| C_14:0_ | 1.29 |
| **C_16:0_** | **26.07** |
| C_17:0_ | 0.52 |
| C_18:0_ | 0.70 |
| C_19:0_ | 0.35 |
| Hydroxy Fatty acids | |
| C_10:0_ 3OH | 3.01 |
| C_12:0_ 2OH | 3.27 |
| C_12:1_ 3OH | 0.20 |
| C_12:0_ 3OH | 3.31 |
| C_15:0_ 2OH | 0.32 |
| C_18:1_ 2OH | 0.44 |
| C_18: 0_ 3OH | 0.39 |
| Branched Fatty acids | |
| *iso*- C_14:0_ | 0.49 |
| *iso* C_15:0_ | 0.49 |
| *anteiso*- C_15:0_ | 1.32 |
| *iso* C_16:0_ | 0.29 |
| *iso*-C_17:0_ | 0.47 |
| *anteiso*-C_17:0_ | 0.48 |
| Unsaturated Fatty acids | |
| C_17:1_ w8C | 0.28 |
| **C_17:0_ cyclo** | **6.21** |
| C_19:0_ cyclo w8c | 1.03 |
| Summed Feature 3 | 25.56 |
| Summed Feature 5 | 0.52 |
| Summed Feature 8 | 18.98 |

The Summed feature 3 comprises unknown fatty acid C_16:1_w6c or C_16:1_w7c, Summed feature 5 comprises C_18:2_w6c or C_18:2_w9c, *anteiso*- C_18:0_; While summed feature 8 comprises C_18:1_ w7C or C_18:1_ w6C. Analysis has been conducted in triplicates. The predominant components of fatty acids are highlighted in bold.

**Table S3.** Identification of root associated, ACC deaminase producing *Pseudomonas* sp. strain S3 by means of fatty acid methyl ester (FAME) Analysis and partial sequencing of 16S rRNA gene

| Strain | FAME Analysis | | Molecular characterization | | |
| --- | --- | --- | --- | --- | --- |
|  | MIDI Identification | Similarity Index | Closest Match | Similarity | GenBank Accession Number |
| S3 | *Pseudomonas-putida-biotype A* | 0.556 | *Pseudomonas* sp. strain BML3 | 96.03% | MK680061.1 |
